# Revolutionizing urban mapping: deep learning and data fusion strategies for accurate building footprint segmentation

**DOI:** 10.1038/s41598-024-64231-0

**Published:** 2024-06-12

**Authors:** P. Dabove, M. Daud, L. Olivotto

**Affiliations:** 1 Department of Environment, Land and Infrastructure Engineering, Politecnico di Torino, Turin, Italy; 2 DigiSky S.R.L., Turin, Italy

**Keywords:** Segmentation, Building footprint, Data fusion, Urban planning, Deep learning, Civil engineering, Environmental sciences

## Abstract

In the dynamic urban landscape, understanding the distribution of buildings is paramount. Extracting and delineating building footprints from high-resolution images, captured by aerial platforms or satellites, is essential but challenging to accomplish manually, due to the abundance of high-resolution data. Automation becomes imperative, yet it introduces complexities related to handling diverse data sources and the computational demands of advanced algorithms. The innovative solution proposed in this paper addresses some intricate challenges occurring when integrating deep learning and data fusion on Earth Observed imagery. By merging RGB orthophotos with Digital Surface Models, deriving from the same aerial high-resolution surveys, an integrated consistent four-band dataset is generated. This unified approach, focused on the extraction of height information through stereoscopy utilizing a singular source, facilitates enhanced pixel-to-pixel data fusion. Employing DeepLabv3 algorithms, a state-of-the-art semantic segmentation network for multi-scale context, pixel-based segmentation on the integrated dataset was performed, excelling in capturing intricate details, particularly when enhanced by the additional height information deriving from the Digital Surface Models acquired over urban landscapes. Evaluation over a 21 km^2^ area in Turin, Italy, featuring diverse building frameworks, showcases how the proposed approach leads towards superior accuracy levels and building boundary refinement. Notably, the methodology discussed in the present article, significantly reduces training time compared to conventional approaches like U-Net, overcoming inherent challenges in high-resolution data automation. By establishing the effectiveness of leveraging DeepLabv3 algorithms on an integrated dataset for precise building footprint segmentation, the present contribution holds promise for applications in 3D modelling, Change detection and urban planning. An approach favouring the application of deep learning strategies on integrated high-resolution datasets can then guide decision-making processes facilitating urban management tasks.

## Introduction

Building footprint segmentation is the process of identifying and outlining the exact location and shape of buildings from aerial or satellite imagery. Building footprints are crucial in various applications, including urban planning, infrastructure management, and land use analysis^1^. In the context of urban planning, they offer valuable insights into building distribution, facilitating efficient land use and development strategies^2^. Accurate segmentation of building footprints is equally essential for change detection, enabling the identification of new constructions, demolitions, or building alterations over time^3^.

Beyond urban planning, precise building footprint segmentation holds significance in natural hazard management and digital twin model development^4^. It contributes to vulnerability assessments, guides disaster response strategies, and enhances the fidelity of digital twins for realistic simulations^5^. Furthermore, its applications extend to 3D city modelling and scene perception, supporting the creation of lifelike visualizations and immersive virtual environments within digital twins.

The proliferation of high-resolution orthophotos captured by airborne vehicles, commercial satellites, and unmanned devices has made manual building segmentation impractical due to the challenges posed by substantial data volumes. Consequently, exploring automation processes has become imperative, although not without challenges such as occlusions, diverse building types, and the complexity of urban landscapes. This shift towards automation has become particularly relevant as automatic methods for Building Footprint Segmentation can be broadly categorised into three fields.

Rule-based methods^6^ rely on predefined criteria and heuristics, while machine learning methods, such as support vector machines^7^ and random forests^8,9^, use labelled training data for automatic learning and identification of building structures. Nevertheless, limitations can be encountered when using the above-mentioned methods. While rule-based approaches struggle in complex urban landscapes and varying building types, machine learning methods demand extensive labeled data facing newer challenges when being adapted to new environments.

The rise of deep learning techniques^10^, particularly convolutional neural networks, has showcased remarkable capabilities in capturing complex spatial patterns and semantic information from visual data, making them well-suited for building footprint segmentation. Despite their effectiveness, most deep learning methods primarily leverage RGB imagery, limiting their ability to capture crucial height information in urban contexts. To address this limitation, some approaches incorporate multi-source data, such as LIDAR^11–15^, synthetic aperture radar^16^, or multi-spectral imagery^17^. However, adopting multi-source data as a strategy for building footprint segmentation poses notable challenges, encompassing elevated data acquisition costs, intricate data registration complexities, and the imperative demand for algorithm calibration.

Alternatively, adopting sophisticated advanced algorithms emerges as a potential avenue to improve segmentation results; however, the present, frequently leads to greater computational requirements and extended processing times. Despite the potential improvement in accuracy, these intricate methodologies counter substantial tasks related to computational resources and time efficiency^18^. Against the increasing prevalence of high-resolution orthophotos, a critical demand arises for methods aimed towards efficient management of large-scale data without compromising either accuracy or integrity.

Facing the above challenges, our approach emphasizes the essential balance between segmentation enhancement and practicality. By integrating various data sources, our method aims to overcome traditional limitations, achieving superior accuracy in building footprint segmentation. This highlights the crucial need to advance algorithms and data fusion techniques for more resource-efficient solutions in urban environment analysis.

This paper proposes a novel method to address the challenges of building footprint segmentation in urban environments. By focusing on improving the quality of data, a unique method has been developed to enhance the extraction of accurate and efficient results in complex surroundings, mainly pointing in two directions:

1. The choice of the dataset integrating data acquired from aerial surveys
2. The selection of the segmentation algorithm.

In the first step, high-resolution RGB orthomosaics and Digital Surface Models were combined into a four-band integrated dataset. Using stereoscopy allows the creation of high-quality Digital Elevation Models from RGB imagery, resulting in consistent and unified datasets obtained from a single source. DSM, which stands for Digital Surface Model^19^, offer elevation details which can significantly improve the contextual understanding of natural and artificial structures. This advancement eliminates the need for additional data sources, such as LIDAR or multi-spectral optical sensors, to be disposed of which could introduce additional issues. The high-resolution data produces a high-quality DSM offering detailed elevation information significantly enhancing the understanding of the built structures and their related surroundings.

On the other hand, the application of the DeepLabv3 algorithm, designed explicitly for multi-scale context, excelled in capturing fine details. DeepLabv3^20^ is an advanced architecture utilising atrous convolution and atrous spatial pyramid pooling (ASPP) modules to capture multi-scale contextual information and refine object boundaries becoming a well-suited tool when working with the integrated RGB + DSM datasets. Having compared the performance of DeepLabv3 with the traditional UNET^21^ algorithm, the improvement achieved by this novel methodology has been demonstrated.

The study area used for evaluation spans 21 km^2^ over the city of Turin, Italy, encompassing diverse building types and a complex urban landscape. The current environment has specifically been chosen as it allows us to thoroughly test the effectiveness of the described approach. A 25 cm/pixel RGB orthomosaic and 50 cm/pixel Digital Surface Model collected by DigiSky S.r.l. Company have been the input data for the cited analysis. To efficiently measure the models’ performance, the recall, F1 score, and IoU techniques have been considered and analyzed. The results demonstrate the superiority of the proposed method in terms of accuracy and boundary refinement, showcasing the potential for practical applications in 3D modelling, change detection, and urban planning. This approach offers a promising solution for precise building footprint segmentation of built environments by leveraging high-resolution integrated datasets and state-of-the-art segmentation algorithms. The combination of multi-channel data fusion and elevation information significantly enhances the accuracy and efficiency of the segmentation process, paving the way for improved urban planning and infrastructure management strategies.

Summarizing, there are two main novelties in this research paper:

1. While overcoming the limitations of multi-source data integration challenges, the proposed method focuses on a singular yet robust source of data using stereoscopy, streamlining the process and mitigating diverse data acquisition costs, registration challenges, and algorithm calibration complexities.
2. Eliminating the need for highly complex algorithms in multi-source fusion, this approach simplifies the computational demands and processing time while maintaining effectiveness in building footprint segmentation at the highest quality.

## Related work

The building footprint segmentation literature encompasses three primary domains. Rule-based methods rely on predefined rules and thresholds, machine learning employs algorithms used for image classification based on feature extraction, and deep learning utilises convolutional neural networks. Furthermore, data fusion integrates diverse sources to enhance precision during the building segmentation process.

### Rule-based approaches

Within the domain of building parcelling methodologies, rule-based approaches have conventionally leaned on pre-established rules or thresholds, leveraging spectral or geometric features for segmentation^6,22^. A pivotal historical exploration carried out in 1988 by Huertas and Nevatia, outlined a methodology to be used for building detection applied to aerial images. This method, rooted in edge detection, shadow analysis utilizing the direction of illumination, and shape analysis employing rectangular models to represent buildings, facilitated the segmentation of the built differentiating it from the surrounding environment^23^.

Rule-based building detection faces adaptability and accuracy disputes in high-resolution optical remote sensing. Diverse urban structures lead to errors, as evident in the Vaihingen 2D Labeling Contest^24^ where rule-based methods underperformed compared to deep learning strategies. Their limited adaptability and reliance on simplistic models made them less favourable, but their potential as post-processing supplements for sophisticated methodologies has been acknowledged^25^.

### Machine learning approaches

Recently, machine learning has become an integral approach in building detection from remotely sensed orthophotos, employing various supervised and unsupervised algorithms for pixel or object-based classification. These computational methods can be based on features like colour, texture, or shape^26^. Various classifiers, such as a support vector machine (SVM), have been used, for instance, for texture-based aerial image segmentation^7^. A random forest (RF) classifier for spectral-based structure segmentation, instead, has been explored when operating on satellite images^8,9^. Building upon this foundation, a research investigation explored the integration of DSM with orthophotos by applying five distinct algorithms, revealing the random forest algorithm as the most-performing method^9^.

Furthermore, the integration of LiDAR data with high-resolution imagery has been examined in the past to enhance feature representation for building extraction. Utilising a building extraction layer with high-resolution imagery (HRI) data sees random forest classification being employed for adequate building type distinction in urban areas. However, challenges persist when harmonizing diverse data sources and when managing computational demands for processing multidimensional data^12^. Ongoing obstacles in the field include point cloud sparsity, high spectral variability, urban object differences, surrounding complexity, and data misalignment^11^. Feature selection or extraction problems may, furthermore, hinder machine-learning approaches^27^. The complexity of building footprints in traditional orthophotos can challenge model learning, leading to segmentation inaccuracies requiring a substantial amount of variables^9^. Factors like relief displacement causing misalignment between the roof outline and the actual building footprint, especially for high-rise buildings, introduce complexity impacting the learning capability of segmentation models^3^. Addressing these challenges becomes crucial for the advancement of building detection and segmentation applications in complex urban environments.

### Deep learning approaches

In the realm of building footprint segmentation, deep learning approaches employing convolutional neural networks (CNNs) have become pivotal, showcasing remarkable capabilities in pixel-based or object-based semantic segmentation on orthophotos^10,28,29^. The extensive array of deep learning algorithms, including AlexNet, fully convolutional networks, U-Net, VGG, GoogLeNet, ResNet, DenseNet, LinkNet, pyramid scene parsing network, bottom-up and top-down feature pyramid network, and DeepLabv3 and DeepLabv3+, have demonstrated their efficiency in achieving both accuracy and robustness during the building footprint segmentation process^30^.

While Mask R-CNN combined with building boundary regularization enhances the refinement of building polygons, the generalization ability to other contexts remains limited^31^. Incorporating multi-source data, such as very high-resolution aerial imagery and multi-source GIS data, introduces challenges and opportunities, requiring careful consideration to achieve optimal results^32^.

An approach where RGB orthophotos are used as the primary input in most deep-learning processes overlooks the enrichment which elevation information can bring especially if derived from multi-sources. Conversely, the richness of details within multi-source data poses challenges in developing accurate deep-learning models for building footprint extraction^33^. DeepLabv3, known for its edge precision and multi-scale context, can offer advantages when applied to a combination of RGB and DSM data, as highlighted by MAP-Net’s comparison^34,35^.

While transformers have shown promise in building detection and segmentation tasks, there are limitations to consider. For instance, the complexity of transformer models may lead to increased computational requirements and training times^36^. Additionally, transformers may struggle with capturing fine-grained details in building structures, especially in scenarios with limited data or diverse building types^37^.

The evaluation of deep learning-based methods, used as a discriminant factor between buildings and the background, traditionally prioritizes metrics ensuring the extraction of the bulk of building footprints. However, these metrics are not yet fully able to address the computational time and resource requirements, emphasizing a comprehensive assessment framework^38^. Applying deep learning models in remote sensing for building extraction tasks has inspired several researchers guiding them towards the exploration of advanced techniques capable of addressing the computational complexity inherent in such tasks^1^.

### Data fusion approaches

Data fusion combines data coming from different sources to create a new dataset able to provide greater quality information than their respective sources. Data fusion can be performed at different levels, such as pixel, feature, or decision levels^39^. In this paper, pixel-level data fusion has been the focus, combining the pixel values of different images to create a new dataset with more bands or a higher resolution^40^. Data fusion with elevation information can therefore improve building footprint segmentation by enhancing the contrast between buildings and backgrounds aiding the segmentation process of building boundaries.

Historically, many methods using DSM data for building extraction did not incorporate RGB data, which limited their effectiveness. One study^41^ used a two-step global optimization process on DSMs but faced challenges with low-rise and non-rectilinear buildings. Tian et al.^42^ used DSMs for urban change detection, relying on height information and Kullback–Leibler divergence measures, yet lacked the contextual richness that RGB data could provide. Bittner et al.^43^ applied a Fully Convolutional Network (FCN) to DSMs for building mask extraction, which could have been enhanced with RGB data for better material classification and feature extraction.

In contrast, a recent study^44^, integrated RGB data with DSMs and the Visible-band Difference Vegetation Index (VDVI), significantly improving the accuracy of building extraction, especially in complex areas where buildings are obscured by vegetation. This fusion allowed for better differentiation between buildings and ground objects. Despite these advancements, the evolving field of deep learning necessitates more robust algorithms that can capture multi-contextual details to further refine segmentation accuracy in complex urban environments.

The study by Marmanis et al.^45^ examines the improved accuracy in semantic image segmentation of man-made structures using boundary detection while recognizing the challenges faced when processing vegetation classes. A nuanced approach is then proposed as it may impact the result generalization in urban environments during data fusion scenarios.

Additional studies^15^ highlight a significant achievement, throughout the successful fusion of both aerial imagery and LiDAR data, obtained in an active contour segmentation algorithm application. However, once again, multi-source data presents tremendous tasks, such as ensuring compatibility between different data formats and the need for calibration front variations in resolution and accuracy, which comes with data fusion.

Literature also addresses the challenges associated with data fusion techniques. For instance, one study^3^ highlighted the misalignment between roof outlines and building footprints in traditional orthophotos, posing challenges for accurate building footprint extraction, especially for high-rise buildings. Additionally, other evaluations^46^ discussed overcoming missing and incomplete modalities with Generative Adversarial Networks applied to the building footprint segmentation process.

By indicating the complexities involved in fusing diverse data modalities, research^33^ demonstrated that incorporating additional height information improved the overall segmentation quality for building footprint extraction, significantly increasing prediction accuracy.

Data fusion emerges as a pivotal technique in building footprint segmentation, harnessing information from various sources to create enhanced datasets at different levels: pixel, feature, or decision levels^39^. This paper concentrates on pixel-level data fusion, specifically blending pixel values from distinct images to yield a new image having expanded bands or heightened resolution^40^. Highlighting the significance of elevation information in data fusion, particularly with Digital Surface Models (DSM), proves how building footprint segmentation can be enhanced and refined. Studies indicate that the fusion of RGB and DSM orthophotos outperforms RGB orthophotos alone, emphasizing improved accuracy and boundary delineation^32^.

Despite the progress made in data fusion techniques, challenges persist. Integrating multi-source data, as demonstrated in the studies incorporating RGB with LiDAR data or employing advanced algorithms like the Gated Residual Refinement Network, presents hurdles such as compatibility issues, resolution variations, and the need for extensive labelled datasets for training^13,47^. Notably, traditional orthophotos face misalignment challenges between roof outlines and building footprints, impacting accurate extraction, especially for high-rise buildings^3^. The complexity is further magnified by missing and incomplete modalities, prompting innovative solutions, such as Generative Adversarial Networks, to be deployed for building footprint segmentation^46^.

In navigating these challenges, recent studies underscore the transformative potential of incorporating additional height information in the fusion process. The integration of height data enhances the overall segmentation quality and significantly boosts prediction accuracy^33^. As building footprint segmentation continues to evolve, the judicious exploration of data fusion methodologies, their challenges, and innovative solutions stand at the forefront, driving advancements in urban planning and within the change detection field.

## Methodology

In this section, the methodology adopted by the current study is treated, including the used data sources, data fusion techniques, deep learning algorithms deployed, and evaluation metrics adopted.

### Dataset description

**Figure 1:**
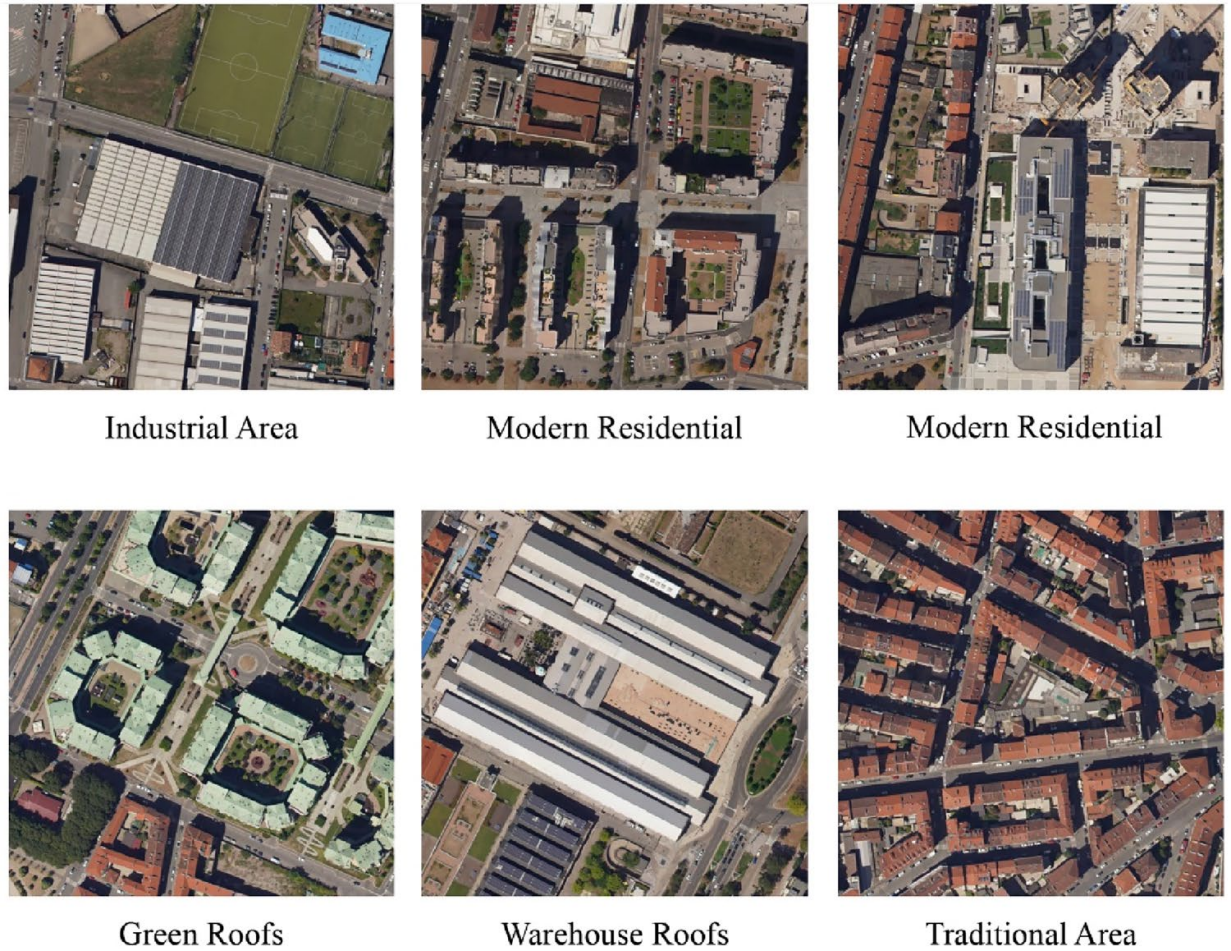
Example of different roof types in the case study.

The two basic raster layers used in this study, have been acquired by aerial photogrammetry campaigns carried out by DigiSky S.r.l. Company. The primary input raster files adopted are described as follows:

- *RGB Orthomosaic*: This raster holds three bands (Red, Green, and Blue) exhibiting a ground sampling resolution of 25 cm/pix (Fig. 1). It provides spectral information for building footprint segmentation.
- *DSM raster layer*: This raster file contains one band elevation information holding a resolution of 50 cm/pixel. It was derived from stereoscopic triangulation processes applied to photogrammetric aerial images. It also provides elevation information for building footprint segmentation.

### Data fusion processing

**Figure 2:**
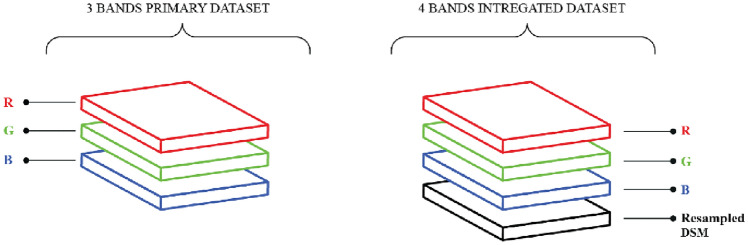
Schematic diagram of the data fusion process.

This paper employs pixel-level data fusion, combining RGB and DSM Orthomosaics to create a four-band integrated dataset. This fusion (Fig. 2) enhances spectral and elevation information crucial for precise building footprint segmentation. The fusion process consisted of the following steps:

- *Resampling* the DSM raster layer had been resampled from 50 to 25 cm resolution using the nearest neighbour method to match the resolution of the RGB Orthomosaic.
- *Cropping* RGB and DSM Orthomosaics were both cropped to the same extent and size while holding the extent of the RGB Orthomosaic as a reference.
- *Stacking* RGB and DSM layers have been stacked along the band dimension to create a new dataset with four bands: Red, Green, Blue, and Elevation.
- *Normalization* pixel values were normalized as each band featured values ranging from [0, 1] using min–max normalization.

**Figure 3:**
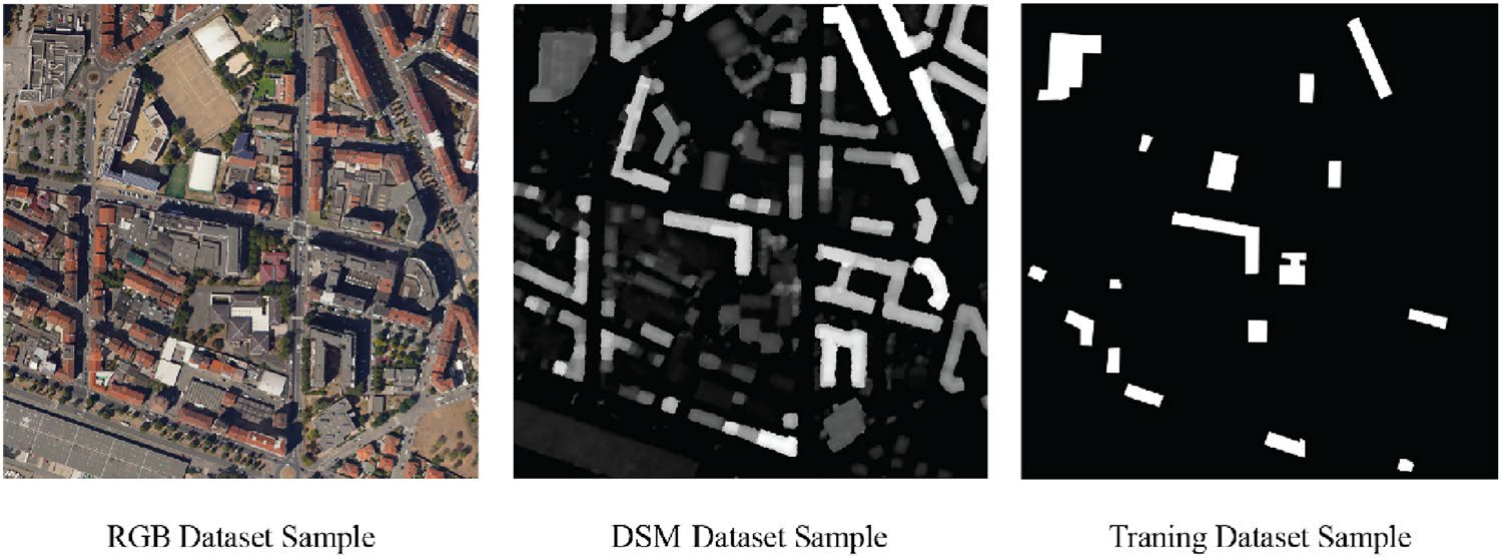
Side-by-side comparison of RGB, DSM, and training dataset.

The data fusion process enhanced the contrast between buildings and backgrounds strengthening the segmentation process of the building boundaries. Figure 3 displays a simple side-by-side comparison of the RGB, DSM, and the training dataset utilized for the present study. It clearly illustrates how the DSM influences boundary delineation, as shown in the binary image.

The two inputs mentioned above were used to create datasets for building footprint segmentation analysis:

- *Primary dataset*: This dataset originated using the RGB Orthomosaic (3 Bands). It provides only spectral information for building footprint segmentation.
- *Integrated dataset*: This dataset combines the RGB and DSM Orthomosaics (3 + 1 Bands). It provides both spectral and elevation information for building footprint segmentation.

### Deep learning algorithms

This study employs U-Net and DeepLabv3, two leading deep learning algorithms utilizing convolutional neural networks for pixel-based semantic segmentation on orthophotos. Assigning class labels (building or non-building) to individual pixels results in one of the focal points of this article. With an extensive analysis, a comparison between the performance of these algorithms for both standalone and integrated datasets is carried out, evaluating accuracy and boundary delineation characteristics.

#### U-Net

**Figure 4:**
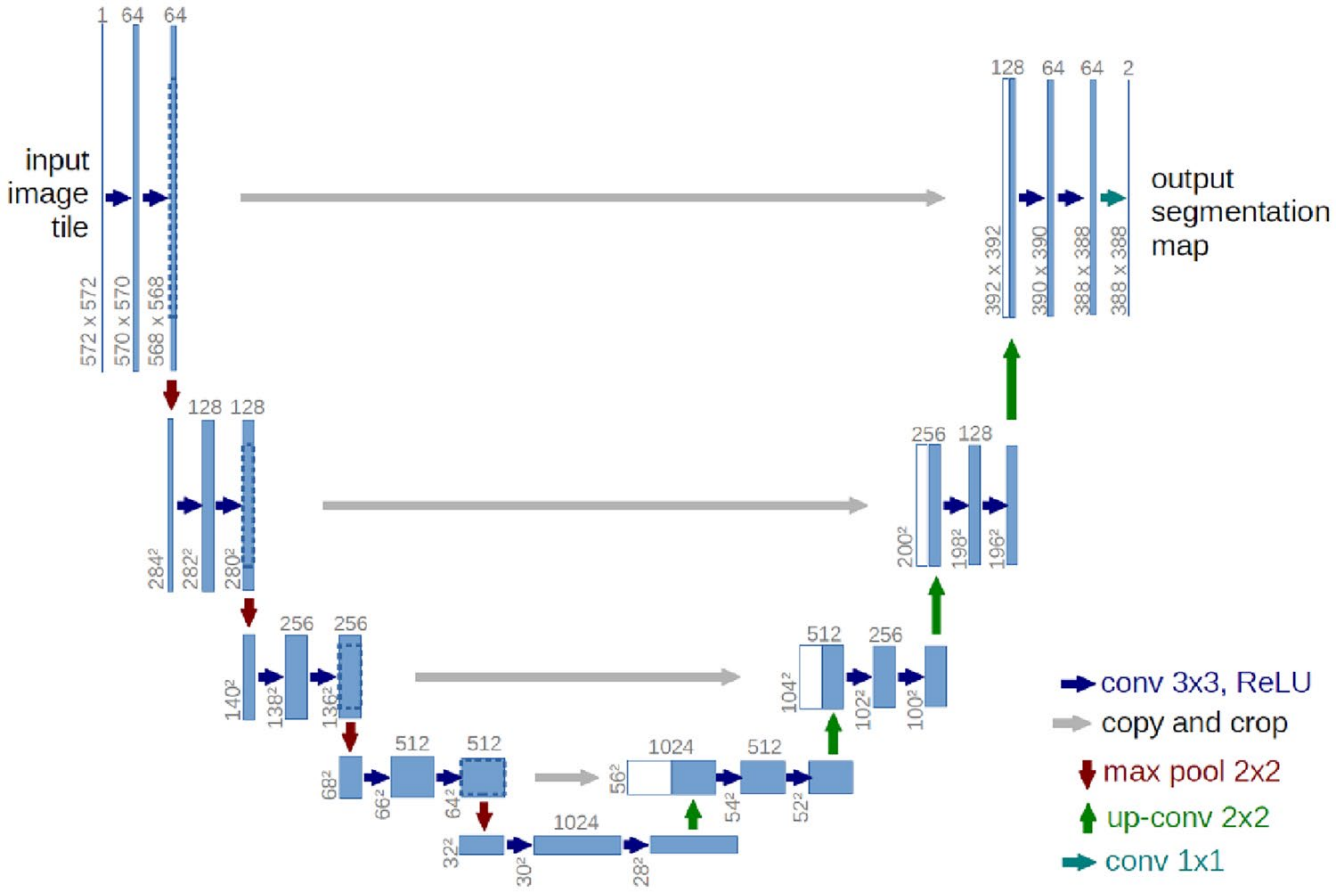
Architecture of U-Net^21^.

U-Net is a famous encoder–decoder architecture using skip connections to recover spatial information from low-level features. The encoder consists of convolutional layers which progressively reduce the spatial resolution whilst increasing the feature dimension. The decoder includes deconvolutional layers that progressively increase the spatial resolution and decrease the feature dimension. The skip connections link the encoder and decoder layers at corresponding resolutions concatenating their features. The output layer generates a pixel-wise prediction map exhibiting the same resolution as the input image. Figure 4 describes the architecture of the encoder–decoder of the U-Net Algorithm^21^:

U-Net presents several advantages for building footprint segmentation. These can be synthesized as follows:

- Variable input sizes are handled producing output maps with high resolution.
- Capability of capturing both local and global features from different levels of abstraction.
- Fine-grained detail recovery starting from low-level features using skip connections.

However, U-Net also presents some limitations:

- Due to its fixed kernel size, it may not capture sufficient contextual information from large receptive fields.
- Its bilinear interpolation in deconvolutional layers can produce blurry or inaccurate boundaries.

#### DeepLabv3

DeepLabv3 is an advanced architecture using atrous convolution and atrous spatial pyramid pooling (ASPP) modules to capture multi-scale contextual information refining object boundaries. Atrous convolution allows the adjustment of the effective field of view of convolutional filters without changing their size or number of parameters^48^. ASPP is, therefore, a technique that applies atrous convolution with different rates to capture features at multiple scales.

**Figure 5:**
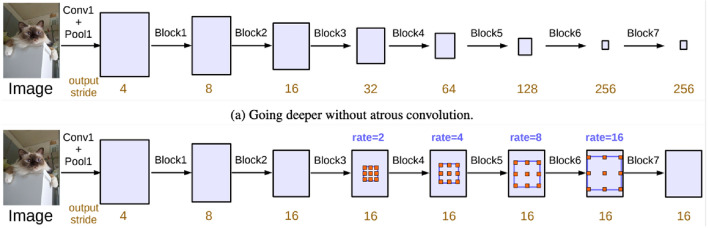
Architecture of DeepLabv3^20^.

Figure 5 defines the architecture of the encoder-decoder of the U-Net Algorithm along with atrous convolution^20^:

DeepLabv3 presents several advantages for building footprint segmentation, such as:

- It can handle large receptive fields efficiently using atrous convolution.
- It can capture multi-scale contextual information using ASPP modules.
- It can refine object boundaries using atrous rates that match object scales.

Despite the advantages, DeepLabv3 also introduces some limitations, such as:

- It may produce output maps with lower resolution than U-Net algorithms due to its down-sampling operations, requiring a binary classification in building footprint segmentation.

### Training and validation data processing

**Figure 6:**
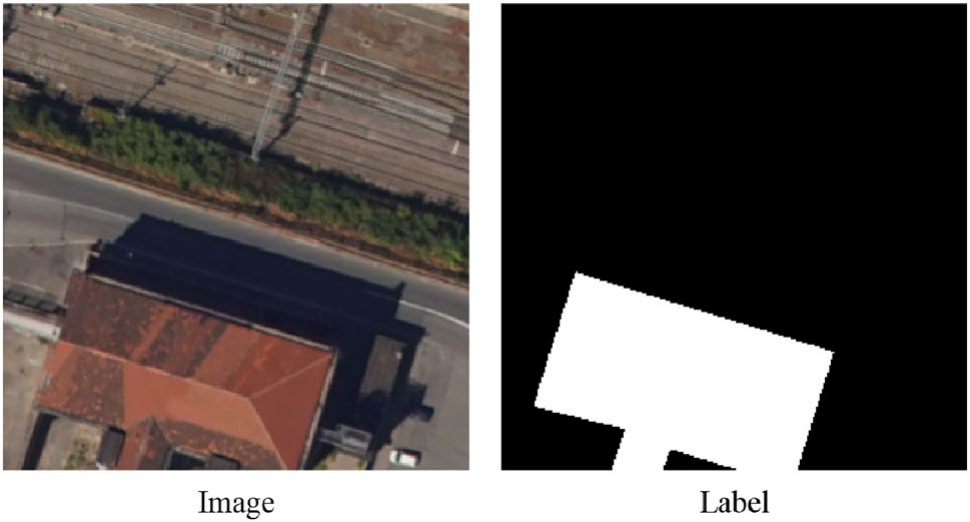
Example of a binary mask.

During the data preparation phase, four hundred and fifty buildings were manually digitized using ArcGIS Pro software, relying on visual inspections of the RGB Orthomosaic. This dataset was thoughtfully cured to encompass various building characteristics, including varying sizes, shapes, roof types and orientations within the urban context. To facilitate the deep learning process, the digitized building footprints were converted into binary masks, each with a pixel size of 256 × 256—a binary value of 1 represented by building pixels, while 0 denoted background pixels. Refer to Fig. 6 for an illustration of a sample binary mask.

The dataset was then partitioned into an 80% training set and a 20% validation set, maintaining the same repartition for both the primary and integrated datasets.

Subsequently, for the training and validation processing stages, TensorFlow has been employed as the reference deep learning framework. Framework complemented by the ArcGIS Pro 3.1 deep learning libraries used for tasks such as exporting tiles/masks, visualization, and sample preparation. The selected hardware configuration, featuring a 12 GB GDDR6X GPU RTX 3080, an Intel Core i7 9th generation CPU system, and 16 GB of System RAM, was specifically tailored to enhance the efficiency of the training and validation processes (Table 1).

**Table 1:** Flops and params for the models.

	Flops	Parameters (M)
U-Net primary	84.53B	36.4
DeepLabv3 primary	50.08B	37.1
U-Net integrated	104.41B	36.5
DeepLabv3 integrated	62.60B	37.2

Following are the parameters and flops:

Critical configurations for training included:

1. Utilization of the SoftMax activation function and cross-entropy loss function for pixel-wise classification.
2. Implementation of the Adam optimizer with a learning rate of 0.001 and a decay rate of 0.0001 for gradient descent.
3. Adoption of a batch size of 8 with 20 epochs for training. A stride of 128 pixels for sliding window inference.
4. Incorporation of the orthogonal rotation technique for data augmentation.

**Figure 7:**
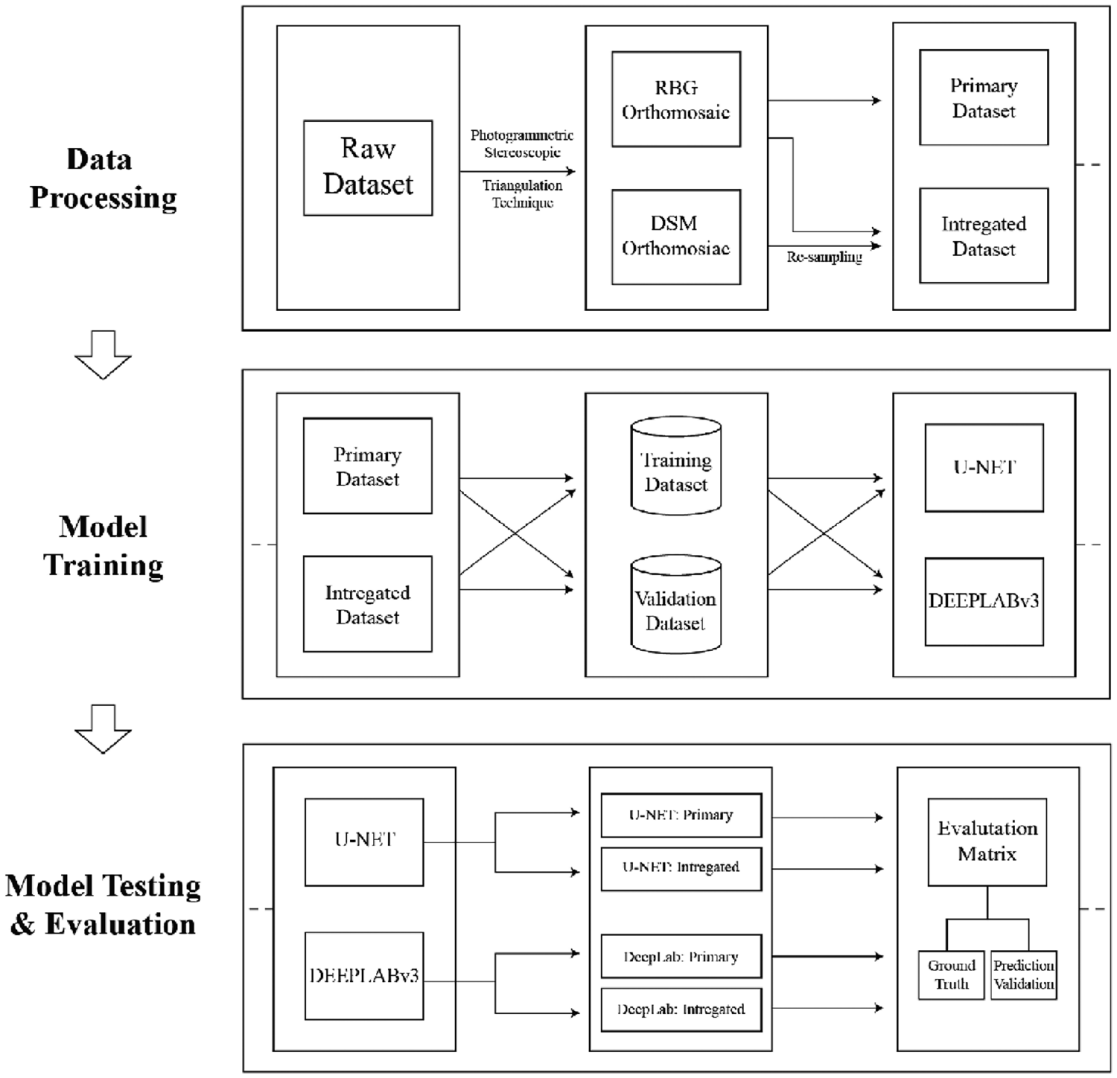
Workflow of a study.

Both U-Net and DeepLabv3 architectures utilize ResNet-50 as their backbone, leveraging its renowned skip connections to address the degradation problem affecting deep networks^49^. This structure enhances feature extraction capabilities for accurate and rapid image segmentation across diverse datasets and tasks. The overall workflow diagram of the process is depicted in Fig. 7.

## Results and analysis

The influence of data fusion and elevation information on building footprint segmentation will be examined in the upcoming sections using different evaluation metrics.

### Evaluation metrics

Various result metrics have been considered and analyzed to assess our models’ performance for building footprint segmentation. These metrics are based on the confusion matrix (shown in Table 2), a table highlighting the number of true positives (TP), false positives (FP), true negatives (TN), and false negatives (FN) for each class (building or non-building).

**Table 2:** Confusion matrix.

	Actual building	Actual non-building
Predicted building	TP	FP
Predicted non-building	FN	TN

The result metrics considered have been listed as follows:

- *Precision* The ratio of correctly predicted building pixels to the total number of predicted building pixels. It measures how precise the model responds during the identification of building pixels.
  $$\text{Precision }=\frac{TP}{TP+FP}.$$
- *Accuracy* The ratio of correctly predicted pixels to the total number of pixels. It measures the accuracy of the model while identifying building and non-building pixels.
  $$\text{Accuracy }=\frac{TP+TN}{TP+TN+FP+FN}.$$
- *Recall* The ratio between correctly predicted building pixels to the total number of actual building pixels. It measures how complete the model is in the identification of building pixels.
  $$\text{Recall }=\frac{TP}{TP+FN}.$$
- *F1 score* The harmonic mean of precision and recall. It measures the balance between precision and recall. It provides an overall look at how our model is performing and its sensitivity to precision and recall criteria.
  $${F}_{1}=2\cdot \frac{\text{ Precision }\cdot \text{ Recall }}{\text{ Precision }+\text{ Recall }}.$$
- *IoU (intersection over union)* IoU measures the spatial agreement between predicted and actual building pixels. It is the ratio of the overlap area to the total area. Higher IoU indicates accurate segmentation.
  $${IoU}=\frac{\text{ TP }}{\text{ TP+FP+FN }}.$$

To provide a more complete analysis, the time necessary to train 20 epochs for each model-dataset combination was reported, reflecting the computational efficiency of the models.

### Performance evaluation

Table 3 reports the result metrics for each algorithm-dataset combination and the time required to train 20 epochs.

**Table 3:** Result metrics for each algorithm-dataset combination.

	Precision	Recall	F1	IoU	Time to train 20 epochs
U-Net primary	0.868	0.884	0.876	0.789	8 h 55 min
DeepLabv3 primary	0.914	0.908	0.911	0.847	2 h 48 min
U-Net integrated	0.894	0.891	0.892	0.816	9 h 23 min
DeepLabv3 integrated	0.928	0.925	0.926	0.873	3 h 20 min

**Figure 8:**
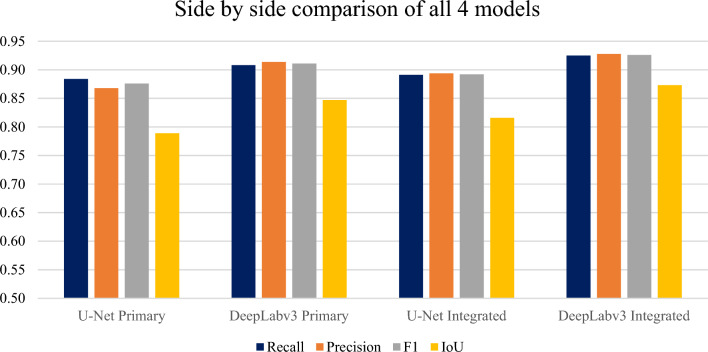
Performance of each algorithm-dataset combination.

The quantitative results presented in Table 3 reveal a nuanced interplay between model architecture, dataset complexity, and computational efficiency in building footprint segmentation. DeepLabv3 Integrated emerged as the top performer, achieving a 0.925 recall, 0.926 F1 score, and 0.873 IoU. This represents a notable improvement over DeepLabv3 Primary, with increases of 1.9%, 1.6%, and 3.1% respectively. This result is further supported by the visual analysis in Fig. 8, which reveals a clear advantage for DeepLabv3 Integrated across all evaluated metrics.

Both U-Net and DeepLabv3 benefited from the Integrated dataset, with DeepLabv3 showing a more substantial increase (2.2% on average across all metrics) compared to U-Net (1.9%). This suggests that DeepLabv3’s atrous spatial pyramid pooling (ASPP) module, which captures multi-scale contextual information, effectively leverages the additional features in the Integrated dataset, particularly in regions with varying building sizes.

Computationally, DeepLabv3 outperformed U-Net consistently in both datasets. DeepLabv3 Primary required 168 min for 20 epochs, a 68.6% reduction compared to U-Net Primary’s 535 min, due to its efficient use of atrous convolutions. Even with the Integrated dataset, DeepLabv3 Integrated trained in 200 min, 64.4% faster than U-Net Integrated’s 563 min.

The Integrated dataset’s higher complexity (RGB + Digital Surface Model) enhanced the model’s ability to capture intricate details, resulting in improved segmentation performance and higher metrics such as recall, F1 score, and IoU. The proportionally larger increase for DeepLabv3 likely stems from its enhanced ability to process the Integrated dataset’s complex features, offset by substantial gains in metrics, notably a 3.1% improvement in IoU in DeepLabv3 Integrated over DeepLabv3 Primary.

**Figure 9:**
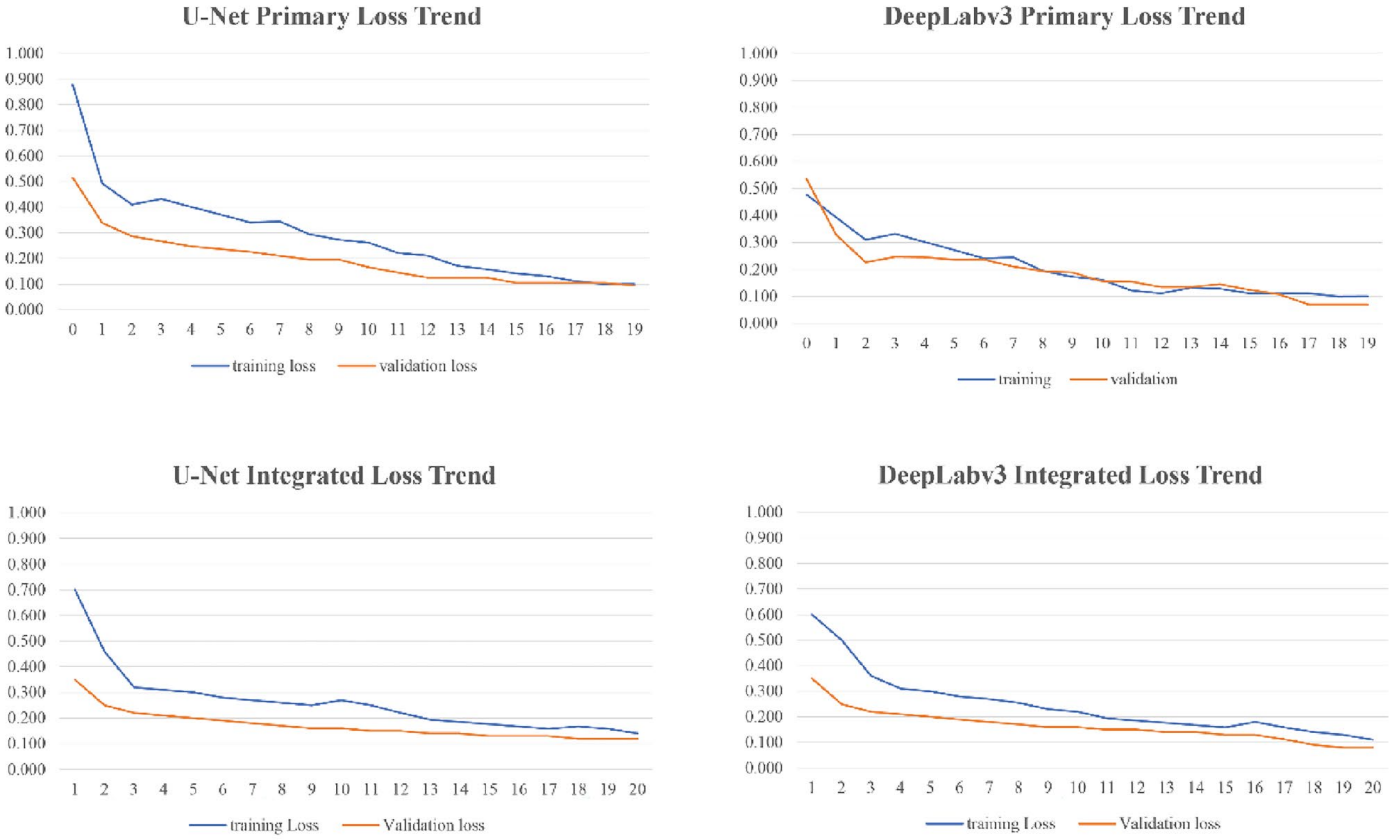
Model complexity and stability of each algorithm-dataset combination.

These observations are supported by the training and validation loss curves in Fig. 9, which show smooth and consistent convergence for the both models around 20 epochs.

These quantitative results underscore the superior performance of DeepLabv3, particularly when coupled with the Integrated dataset. The substantial improvement in all metrics, especially the IoU, coupled with its relative computational efficiency, positions DeepLabv3 Integrated as the optimal choice for building footprint segmentation in this context.

The findings of this study illuminate the pivotal role of data fusion and model architecture in achieving accurate and efficient building footprint segmentation. Leveraging the complementary strengths of RGB and DSM data through the Integrated dataset significantly enhances segmentation performance, particularly for DeepLabv3, which excels at harnessing multi-scale contextual information. While the Integrated dataset introduces a computational overhead, the resulting gains in accuracy, as evidenced by the quantitative metrics, outweigh this trade-off.

These quantitative insights lay the groundwork for a deeper qualitative analysis in the subsequent section. A visual comparison of segmentation outputs across different model and dataset combinations will provide a more nuanced understanding of how data fusion and model architecture impacts the delineation of building footprints in complex urban environments. This qualitative analysis will further illuminate the strengths and weaknesses of each approach, offering valuable guidance for practitioners and researchers seeking to optimize urban mapping workflows.

### Impact of data fusion and elevation information

**Figure 10:**
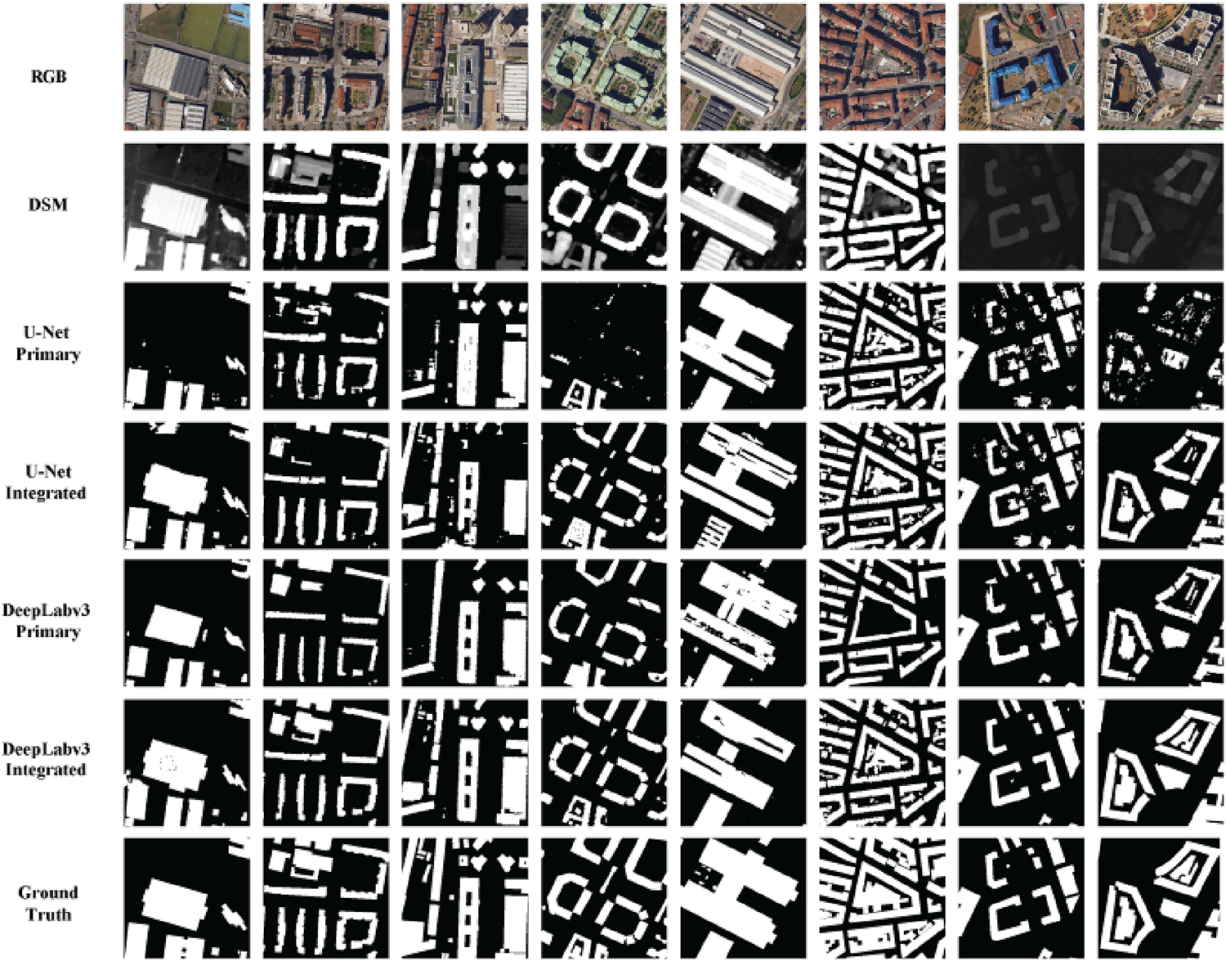
Impact of data fusion and elevation information building footprint segmentation.

Figure 10 provides a comprehensive visual assessment of the impact of data fusion and elevation information on building footprint segmentation across eight diverse and challenging urban scenarios. Each scenario features distinct building typologies, ranging from simple rectangular structures to those with complex shapes, irregular rooftops, and varying surrounding environments. This diversity allows for a nuanced evaluation of model performance under realistic conditions.

The most striking observation is the significant improvement in U-Net’s performance when height information is incorporated. In the first example, U-Net Primary struggles to differentiate closely spaced buildings, leading to under-segmentation and merged rooftops. This is a common challenge in dense urban areas where buildings are tightly packed. However, U-Net Integrated, leveraging the additional height data, clearly delineates individual buildings and avoids the merging error, showcasing the value of elevation information in resolving ambiguities caused by overlapping structures. This improvement is also evident in the third example, where U-Net Primary misclassifies part of a complex rooftop as background due to shadowing effects. The integrated model, however, accurately identifies the entire rooftop, highlighting the role of height information in disambiguating shadowed regions.

Despite the improvements brought by height information, U-Net Integrated is still surpassed by DeepLabv3 Primary in most scenarios. This can be attributed to DeepLabv3’s superior architecture, which incorporates atrous spatial pyramid pooling (ASPP). This module allows the model to capture multi-scale contextual information, enabling it to better understand the complex spatial relationships within the urban scene. In the second example, which features a building with an irregular shape and a courtyard, DeepLabv3 Primary produces a more accurate and continuous outline compared to U-Net Integrated, which struggles to maintain the building’s structural integrity. This indicates that DeepLabv3’s ASPP module is more effective at handling complex geometries and occlusions.

The integration of height information further enhances DeepLabv3’s already impressive performance. This improvement is most evident in the sixth and seventh examples, which depict buildings in areas with varying terrain elevation. In the sixth example, DeepLabv3 Primary misclassifies part of building as ground areas due to the similar spectral signatures of the building and the elevated ground. DeepLabv3 Integrated, however, leverages the height information to accurately distinguish between the building and the terrain, resulting in a cleaner and more precise segmentation mask.

In conclusion, the qualitative analysis presented in Fig. 10 visually reinforces the quantitative findings, offering a comprehensive understanding of how data fusion and model architecture influence building footprint segmentation performance. The figure showcases the superiority of DeepLabv3 Integrated across diverse building typologies and urban environments, highlighting its ability to accurately delineate building footprints even in challenging scenarios. The visual comparison of segmentation outputs not only confirms the quantitative improvements observed but also provides valuable insights into the specific strengths and weaknesses of each model and dataset combination. This qualitative assessment is crucial to understand the nuances of model behaviour while tailoring urban mapping workflows to specific contexts and requirements.

## Discussion

The findings of this study highlight the transformative potential of data fusion in urban mapping, particularly in the context of building footprint segmentation. By synergically combining RGB and DSM data within the Integrated dataset, we have achieved significant improvements in segmentation accuracy, precision, and overall model performance. This is evident not only in the quantitative metrics presented but also in the qualitative visual analysis of Fig. 10, where the Integrated dataset consistently leads to more precise delineation of building boundaries, especially in complex urban environments.

While DeepLabv3 emerges as a superior architecture due to its atrous convolutions and ASPP module, which effectively capture multi-scale contextual information, the impact of the Integrated dataset is consistent across both DeepLabv3 and U-Net models. This demonstrates the generalizability of the data fusion approach and its potential for broader applicability in urban mapping tasks.

### Interpretation of findings

The results unequivocally identify DeepLabv3 Integrated as the most effective algorithm-dataset combination for building footprint segmentation. It consistently outperforms other configurations in terms of recall, F1 score, and IoU, and exhibits a favourable trade-off between accuracy and computational efficiency.

Several factors contribute to the superior performance of DeepLabv3 Integrated:

1. *Data fusion*: The combination of RGB and DSM orthophotos provides a richer feature space for the model to learn from. RGB data captures the spectral characteristics of buildings and their surroundings, while DSM data offers valuable elevation information. This fusion allows the model to better distinguish between buildings and other urban features, particularly in areas with complex roof structures, varying elevations, or shadows.
2. *DeepLabv3 architecture*: The atrous convolutions and ASPP module in DeepLabv3 are particularly adept at capturing multi-scale contextual information. This allows the model to effectively integrate information from different spatial resolutions, leading to more accurate identification and delineation of building boundaries, even in challenging urban landscapes.

While the effectiveness of DeepLabv3 has been demonstrated in previous studies, this research specifically highlights the synergistic impact of data fusion on its performance. The Integrated dataset not only enhances the accuracy and precision of DeepLabv3 but also makes it more robust to variations in building types and urban environments. This finding underscores the importance of considering data fusion strategies, not just as a means to augment existing datasets, but as an integral part of model development and optimization.

### Generalizability and limitations

The proposed method demonstrates potential generalizability to other metropolitan areas with similar building characteristics, urban layouts, and environmental conditions as those found in Turin, Italy. However, several limitations warrant further investigation:

1. *Region-specific challenges*: The method’s performance may degrade in regions with significantly different building typologies or urban environments. While the integration of DSM data generally improves performance in low-contrast scenarios and with complex structures (as evidenced by Fig. 10), extreme cases may still pose challenges. For instance, regions with highly reflective surfaces or dense vegetation cover may require additional data preprocessing or specialized model adaptations.
2. *Dataset limitations*: The testing dataset, while representative of Turin’s urban landscape, may not be sufficiently diverse to fully evaluate the model’s performance across different geographic regions or architectural styles. Furthermore, the manual digitization and visual inspection process, while carefully executed, may still introduce subtle errors or biases in the ground truth data. Future work should focus on expanding the dataset to include a wider range of urban environments and exploring automated or semi-automated labelling techniques to enhance the quality and diversity of ground truth data.
3. *Alternative architectures*: While U-Net and DeepLabv3 provide a strong foundation for building footprint segmentation, exploring alternative architectures could further improve performance. For instance, models like HRNet, which maintains high-resolution representations throughout the network, or PSPNet, which utilizes pyramid pooling modules to capture global context, may offer advantages in handling fine-grained details and complex urban scenes.
4. *Granular analysis*: This study focuses on pixel-based segmentation, providing a comprehensive evaluation of overall accuracy. However, a more fine-grained analysis that examines segmentation performance across specific building categories (e.g., residential, commercial, industrial) or attributes (e.g., roof type, height, footprint area) could yield valuable insights for urban planning and management applications.

## Conclusions

This paper proposed a new approach combining deep learning and data fusion for accurate building footprint segmentation. The method analysed utilizes RGB orthomosaics and Digital Surface Models creating a comprehensive dataset with spectral and elevation information. The performance using U-Net and DeepLabv3 was evaluated, showing improved accuracy and boundary delineation compared to existing methods. Findings highlight the benefits of data fusion and the contextual information captured by DeepLabv3. Accurate building footprints have significant implications for urban planning and infrastructure management. However, challenges remain, including generalizability, dataset size, alternative architectures, and fine-grained analysis. Future research should address these limitations and apply the approach to different regions and scales with improved data quality and quantity. We hope our work inspires further advancements in building footprint segmentation using deep learning and data fusion techniques.

## Data Availability

Data acquired in this study are available on request by contacting the corresponding author.
